# A Novel Reformed Reduced Kernel Extreme Learning Machine with RELIEF-F for Classification

**DOI:** 10.1155/2022/4795535

**Published:** 2022-03-24

**Authors:** Zongying Liu, Jiangling Hao, Dongrui Yang, Ghalib Ahmed Tahir, Mingyang Pan

**Affiliations:** ^1^Dalian Maritime University, Faculty of Navigation, No. 1 Linghai Road, Dalian 116085, China; ^2^University of Malaya, Kuala Lumpa 50603, Malaysia

## Abstract

With the exponential growth of the Internet population, scientists and researchers face the large-scale data for processing. However, the traditional algorithms, due to their complex computation, are not suitable for the large-scale data, although they play a vital role in dealing with large-scale data for classification and regression. One of these variants, which is called Reduced Kernel Extreme Learning Machine (Reduced-KELM), is widely used in the classification task and attracts attention from researchers due to its superior performance. However, it still has limitations, such as instability of prediction because of the random selection and the redundant training samples and features because of large-scaled input data. This study proposes a novel model called Reformed Reduced Kernel Extreme Learning Machine with RELIEF-F (R-RKELM) for human activity recognition. RELIEF-F is applied to discard the attributes of samples with the negative values in the weights. A new sample selection approach, which is used to further reduce training samples and to replace the random selection part of Reduced-KELM, solves the unstable classification problem in the conventional Reduced-KELM and computation complexity problem. According to experimental results and statistical analysis, our proposed model obtains the best classification performances for human activity data sets than those of the baseline model, with an accuracy of 92.87 % for HAPT, 92.81 % for HARUS, and 86.92 % for Smartphone, respectively.

## 1. Introduction

In recent decades, the rapid advancement in technology increased computation capacity that welcomed the second spring of Artificial Intelligence (AI). As the backbone of AI, Machine Learning (ML) touches our daily life, and even we do not notice. For example, some functions are applied in the wearable devices, including sporting detection, fall detection, and activity detection. These applications based on the classification algorithms are implemented successfully in our real-world life. Many classical neural networks, such as Artificial Neural Network, Support Vector Machine, and Back-propagation algorithm, performed well for the classification tasks [1–3]. However, the main limitation of these algorithms is the heavy computation, especially for the large-scale data. In the support vector machine, the kernel method, which connects the input layer of the model with the hidden layer, increases the computational complexity. At the same time, the main reason of backpropagation and artificial neural network with heavy computation is to compute suitable input weights and output weights for the neural network.

To solve the problem of complex computation, in 2004, Huang et al. proposed a single-layer feed-forward network called Extreme Learning Machine [4]. Due to the random selection of input weights between the input and hidden layer, it was faster thousands of times and achieved better performance in classification than that of the traditional algorithms [5]. After that, Extreme Learning Machine with Kernel (KELM) was proposed [6], which used Gaussian function to connect input layer and hidden layer and then found a least-squares solution. It obtained better performance in classification than that of conventional extreme learning machine [7]. However, the computation of the kernel method is heavy, especially for the large-scale data. In 2016, Deng et al. proposed a fast kernel algorithm called Reduced Kernel Extreme Learning Machine (Reduced-KELM) [8]. It randomly selects a certain percentage of training samples. Although this strategy reduced the computation complexity and solved the limitation of KELM, the random selection method became an unstable element that leads to the unstable forecasting performance.

To overcome the limitations mentioned above, there are two main aims in this study. The first one is to filter out redundant features based on feature selection methods, because the large-scaled data usually appears in the training process. In aspect of feature selection, RELIEF-F is the one of the efficient algorithms that is used to select features in the different models. Paper [9] applied RELIEF-F to select training features for the classifier on the facial expression recognition. Yahdin et al. employed RELIEF-F for the feature selection in the prediction of the relevance education background [10]. In 2021, Cui et al. applied machine learning methods with feature selection method, RELIEF-F, to classify the wood materials [11]. These classification algorithms with RELIEF-F have better performance than those without RELIEF-F. Besides, paper [12] concluded that RELIEF-F had much better performance in feature selection than other feature selection methods. Therefore, RELIEF-F plays a significant role in the feature selection and assisting in enhancing performance of classification.

The other one is to overcome the limitation of random element in the model Reduced-KELM and enhance the performance in the classification. The aim of randomly selected sample in Reduced-KELM is to select samples that represent the all features from the training data. However, random selection approach cannot select all samples with the different features and probably miss important features. This situation leads to the decrease forecasting performance and unstable prediction performance. To solve random influence in the process of selecting training features, clustering method is applied to select suitable samples in the training phase or reduce the complex computation of training part. For example, Wu et al. combined K-means clustering method with KELM. It successfully reduced the complexity of computation in the training process [13]. Huang et al. proposed a clustering method with extreme learning machine for classification, which increased the ability of classification [14]. Moreover, samples selection method also impacts on the model performance. Liu et al. applied samples selection method based on correlation analysis, and Fisher is proposed, which could remove the redundant features that had close correlations with each other, to extreme learning machine [15]. It showed the role of samples selection method in the speech emotion recognition model, which increased the speed of discriminating emotional states of different speakers from speech. These proved that the good samples selection method played a vital role in increase efficient and speed of training model.

Inspired by these summary and conclusions, this study applies RELIEF-F to select reliable features. It discards those insignificant features from the data set, which reduces the computation complexity in the training process. Moreover, a novel sample selection method called Reformed Sample Selection Method (RSSM) is proposed. It takes the advantages of K-means and Correlation Detection Selection (CDS) method and takes new strategy to seek more important samples from the training data by modification of K-means and CDS. This study applies RSSM to successfully replace the random selection part in the conventional Reduced-KELM. The proposed model is called Reformed Reduced Kernel Extreme Learning Machine. It not only solves the limitation of random selection in Reduced-KELM, but also improves the performance in classification. Therefore, the main contributions of this study are summarized as follows:

- RELIEF-F algorithm is applied to select relevant features for the training phase. It directly reduces the computation complexity and has less training time than the baseline model Reduced-KELM.
- We proposed a novel reformed reduced kernel extreme learning machine. It uses an efficient sample selection method to replace the random part of Reduced-KELM and obtain better performance in classification than that of the compared models.
- The proposed model performed better than the baseline models on both benchmark data and real-world data. Especially for human activity recognition, our proposed model has superior ability in human activity recognition task.

This paper is organized as follows.  Section 2 reviews Extreme Learning machine, Kernel Extreme Learning Machine, and the works with RELIEF-F.  Section 3 represents Reduced Kernel Extreme Learning Machine, RELIEF-F for the features reduction, three types of Sample Selection Method (K-means, Correlation Detection Selection, and Reformed Sample Selection Method), and our proposed model.  Section 4 reports on data description, experimental design, the experimental results, and discussion on these results.  Section 5 represents the comparison by the statistical method. Finally, the conclusion is represented in Section 6.

## 2. Related Works

Since the rapid development of machine learning algorithms, the artificial intelligence technologies were applied in various domains and achieved good performance, such as face recognition [16, 17], time series prediction [18, 19], and classification [20, 21]. These algorithms involve some traditional and classical neural networks. Taking Backpropagation Neural Network (BPNN) [22] and Support Vector Machine (SVM) [23] as examples, they showed the superability in classification and regression [24–27]. With the appearance of the ‘big data era,' huge-scale data is collected. However, due to the characteristics of the traditional and classical neural networks, these algorithms cannot afford the heavy computation with large-scale data. The computation cost is a barrier to the implementation of these algorithms in the real world.

In the recent decade, random projection algorithms attracted lots of attention of researchers. Due to the random selection of weights, these types of algorithms solved the heavy computation problem. Extreme Learning Machine (ELM), which was proposed by Huang et al. [4], was one of the random projection algorithms. Paper [28] indicated that ELM was thousands of times faster in the training time and achieved better performance than the traditional neural networks, such as BPNN and SVM in classification and regression. In recent years, this algorithm and its variant algorithms were widely used in many domains, such as the stock market prediction [29], image classification [30], flight control [31], and speech emotion recognition [15].

**Figure 1:**
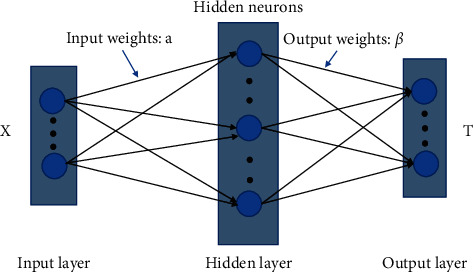
The structure of SLFN.

Because ELM is a modified Single Layer Feed-forward Network (SLFN), before discussing ELM, SLFN should be introduced. The structure of SLFN can be shown in Figure 1, which includes three layers, regarding the input, hidden, and output layers.

We assume that there are N arbitrary samples (*X*, T), where the input samples represent *X* = [*x*_1_, *x*_2_,…, *x*_*L*_,…, *x*_*N*_ ] ∈ *ℜ*^*N*×*W*^, and its corresponding target values are *T* = [*t*_1_, *t*_2_,…, *t*_*L*_,…, *t*_*N*_] ∈ *ℜ*^*N*×*D*^. *L* stands for the number of training samples, and *D* is the number of output nodes. The hidden neurons can be shown as hidden matrix (*H*) that is calculated by the activation function (g (·)). The input weights (*a*) connect between the input layer and hidden layer. Output weights (*β*) connect the hidden layer with the output layer. Then, the output $\left(\hat{T}\right)$ of a feed-forward neural network with S hidden neurons can be expressed as follows:

(1) $$\begin{array}{c} \beta G\left(x\right)=\overset{S}{\underset{i=1}{\sum }}\beta_{i}g\left(a_{i}x+b_{i}\right)=\hat{T}, \end{array}$$

where *S* is the number of hidden neurons, *β* represents the output weights with dimension of (S × D), a is the input weights with dimension of (*S* × *L*), and b is a bias matrix with dimension of (L × S). If there is no error between the activation function g(x) with S hidden neurons in the single-layer feed-forward network and actual target values, the mathematical equation can be shown as follows:

(2) $$\begin{array}{c} \overset{L}{\underset{i=1}{\sum }}\left\|T_{i}-{\hat{T}}_{i}\right\|=0. \end{array}$$

It can be extended as

(3) $$\begin{array}{c} \overset{S}{\underset{i=1}{\sum }}\overset{L}{\underset{j=1}{\sum }}\beta_{i}g\left(a_{i}x_{j}+b_{i}\right)=T_{j}. \end{array}$$

Traditionally, the main aim of training SLFN is the minimization of the cost function for finding the corresponding weights and bias. In this processing, the BP learning algorithm is used from the output to the input. The cost function is shown in

(4) $$\begin{array}{c} E=\overset{L}{\underset{i=1}{\sum }}{\left(\overset{S}{\underset{j=1}{\sum }}\beta_{j}g\left(a_{j}x_{i}+b_{i}\right)-T_{i}\right)}^{2}. \end{array}$$

Unlike SLFN, ELM applies the gradient-based algorithms and proposes an efficient learning algorithm for feed-forward neural networks in order to solve the drawbacks of BP learning algorithm. Based on the theory of ELM, unlike the traditional activation function that requires adjusting the input weights and biases, the input weights and biases of hidden layer can be selected randomly. Then, the training process of ELM is to find a least-squares solution $\hat{\beta }$ of (3), which is shown in the following equation:

(5) $$\begin{array}{c} \left\|\left.H\beta -T\right)\right\|=\underset{\beta }{\min}\left\|\left.H\hat{\beta }-T\right)\right\|, \end{array}$$

where *H* is the hidden matrix based on the activation function. It is a non-squared matrix that can be calculated by (6). The input weights (*a*) and hidden biases *b* were selected randomly.

(6) $$\begin{array}{c} H=\left[\begin{array}{c} h\left(x_{1}\right)\\ \vdots \\ h\left(x_{L}\right) \end{array}\right]=\left[\begin{array}{ccc} g\left(a_{1}x_{1}+b_{1}\right) & \cdots & g\left(a_{S}x_{1}+b_{S}\right)\\ \vdots & \vdots & \ddots \\ g_{\left(a_{1}x_{L}+b_{1}\right)} & \cdots & g\left(a_{S}x_{L}+b_{S}\right) \end{array}\right]. \end{array}$$

Finally, Huang [4] proposed that the smallest norm least-squares solution is

(7) $$\begin{array}{c} \beta =H^{†}T, \end{array}$$

where *H*^†^ represents the Moore-Penrose generalized inverse of matrix *H*. Its mathematical equation can be shown as *H*^†^  =  (*H*^⊺^*H*)^−1^*H*^⊺^, where the superscript (⊺) of *H* stands for the transpose operator. Therefore, the training process can be shown in Algorithm 1.

At the same time, with the advent of the era of big data, the large-scaled data is widely used for training model. However, it also brings the huge computation and decreases the training efficiency. Although the training speed of ELM is faster than that of the conventional algorithms, it also faces this situation. Furthermore, the dimension of training samples impacts the complexity of computation. An efficient filter-method called RELIEF, which was proposed by Kiral [32], showed attributes based on how well their values distinguish among samples that are near each other. After that, Kononenko et al. updated the RELIEF algorithm [33] and proposed the RELIEF-F algorithm. It used the Manhattan (L1) norm to compute the distance between the near-hit and near-miss instances. It reported that RELIEF-F algorithm is an efficient method that takes absolute differences rather than the square of those differences. Besides, to reduce complex computation and increase training efficiency, researchers pay more attention to deal with input features before going through training phase in ELM. For example, Tian et al. applied RELIEF-F as feature selection method in ELM for the gait recognition [34]. In Paper [35], RELIEF-F algorithms is used to propose a feature selection technique for the purpose to eliminate redundancy. It reported that this structure of model with feature selection technique showed significant improvements than other existing forecasting models in terms of forecast accuracy and convergence rate. Many studies [36–39] concluded that RELIEF-F, as a feature selection technique, is an efficient and common approach for eliminating redundant features.

However, due to the random selection of input weights in ELM, the forecasting results are not the same under the same parameters setting of ELM, which causes the unstable forecasting performance, while the number of hidden neurons is also required to define by user. To solve unstable forecasting performance problem, Kernel Extreme Learning Machine (KELM) was proposed by Huang in 2011 [5]. It applied the kernel method to connect input layer and hidden layer, which avoided the unstable forecasting performance from ELM causes by the random selection of the input weights.

In KELM, the hidden matrix (*K*) is calculated by Gaussian function *k*(·), which is represented as

(8) $$\begin{array}{c} K=k\left(X_{L},X_{L}\right), \end{array}$$

where the training samples represent *X*_*L*_  =  {*x*_1_, *x*_2_,…, *x*_*L*_}. The output weights (*β*) of KELM can be computed by

(9) $$\begin{array}{c} \beta ={\left(\text{K}+\frac{\text{I}}{C}\right)}^{-1}{\text{T}}_{L}, \end{array}$$

where *I* is an identity matrix, C represents the regularization parameter that generally is defined as 1, and *T*_*L*_  =  {*T*_1_ is the corresponding training target values (*T*_*L*_  =  {*T*_1_, *T*_2_,…, *T*_*L*_}). The forecasting values $\left(\hat{T}\right)$ can be calculated by

(10) $$\begin{array}{c} \hat{T}=K^{⊺}\beta . \end{array}$$

The papers indicating kernel functions played a vital role in KELM compared with conventional ELM in regression and classification [6, 7, 40]. However, the kernel method with large-scale data generates the huge dimensional kernel matrix, which directly leads to the heavy time consumption in the training process of KELM.

To overcome the limitation of KELM, Deng et al. proposed an efficient and fast model called Reduced Kernel Extreme Learning Machine (Reduced-KELM) [8]. It applied random method to select part of training sample to calculate the hidden kernel matrix, which to some extent reduces the computation. However, due to random selection for training samples, its forecasting performance is not stable. Based on above revision, Table 1 briefly summarizes the advantages and drawbacks of ELM, KELM, and Reduced-KELM.

This study is inspired by the idea of RELIEF-F. Firstly, it applies RELIEF-F to discard useless features of training data. Secondly, to solve the limitation of Reduced-KELM, we propose a novel sample selection method to replace random selection method of Reduced-KELM. Finally, we propose a model named Reformed Reduced Kernel Extreme Learning Machine with RELIEF-F. The following section describes details of the proposed techniques.

## 3. Methodology

This section explains a novel framework for reducing training computation and improving performance during classification. Firstly, RELIEF-F algorithm is proposed for feature selection, which discards the irrelevant features and reduces the training time of the classifier. Secondly, two sample selection methods, including K-mean and correlation detection selection, are introduced. Then, a novel sample selection method named Reformed Sample Selection Method is proposed, which is combined with K-means and Correlation Detection Selection method. Finally, this novel sample selection method successfully replaced the random part of Reduced-KELM, which generates a model called Reformed Reduced Kernel Extreme Learning Machine with RELIEF-F.

### 3.1. Reduced Kernel Extreme Learning Machine

Before describing our proposed methods, the baseline model Reduced-KELM needs to be introduced. The conventional KELM applies all training samples to generate the hidden matrix by Gaussian activation function. The main idea of model Reduced-KELM is to reduce complexity in the computation of kernel matrix by randomly selecting a certain percentage of training samples from all training samples to compute the hidden kernel matrix. It is less time-consuming as it uses only 10 percent of nodes. Paper [8] concluded that Reduced-KELM, randomly selecting ten percentage of nodes, rapidly decreased the training time and achieved almost the same performance as KELM. In the following experiments, we apply ten percent as the random selection percentage in Reduced-KELM.

It is assumed that $\hat{X}$  =  ${\left\{x_{i}\right\}}_{i=1}^{\hat{m}}$ is certain percentage of training samples that are randomly selected, where $\hat{m}$ is the total number of selected samples. Then, the hidden matrix of Reduced-KELM is computed by using the following equation:

(11) $$\begin{array}{c} \hat{K}=k\left(\hat{X},X_{L}\right)=\left[\begin{array}{ccc} k\left(x_{1},x_{1}\right) & \cdots & k\left(x_{\hat{m}},x_{1}\right)\\ \vdots & \vdots & \ddots \\ k\left(x_{1},x_{L}\right) & \cdots & k\left(x_{\hat{m}},x_{L}\right) \end{array}\right]. \end{array}$$

The dimension of the hidden matrix $\left(\hat{K}\right)$ in Reduced-KELM is reduced from (L × L) to (L $\times \;\hat{m}$), which directly decreases the computation of the training process. It computes output weights by

(12) $$\begin{array}{c} \beta ={\left(\frac{I}{C}+{\hat{K}}^{⊺}\hat{K}\right)}^{-1}{\hat{K}}^{⊺}T_{L}. \end{array}$$

The training process of Reduced-KELM is summarized in Algorithm 2.

Reduced-KELM has less training time than the conventional KELM due to the random selection of support vectors for computing kernel matrices. However, the classification result of Reduced-KELM is unstable. To overcome this limitation and to further reduce the training time, this study proposes a RELIEF-F algorithm for selecting features of observations. It represents a novel sample selection method to replace the random selection process of support vectors for enhancing classification performance. The following subsections will introduce the details.

### 3.2. RELIEF-F Algorithm for Features Reduction

In this study, inspired by the characteristic of the RELIEF-F algorithm and successful application on regression and classification models, it is applied to select features from data sets. The following is the process of feature selection by the RELIEF-F algorithm. Firstly, a feature is selected randomly as *R*. Then, its P has searched nearest neighbors from the same class that are named as the nearest hits (*B*). At the same time, it also searches P nearest neighbors from other different classes as *M*. It updates the quality estimation *W*  _*D*_ for all features based on the selected features *R* and *M* by (13). The updated formula is similar to that of RELIEF. Our proposed algorithm weighs the contribution from each class of the misses with the prior probability of that class (P). The contributions of hits and misses in each step will be in the range between zero and one. The values of W determine the ranking of the importance of features. It discards all features with values that are less than zero. The rest of the features continue to process in the training part of the model.

(13) $$\begin{array}{c} W_{D}=\frac{W_{D}-\text{diff}\left\{A,R,B\right\}}{m}+\frac{\sum_{C\neq \text{class}\left(R\right)}\left[P\left(C\right)\text{diff}\left\{A,R,M\right\}\right]}{m}, \end{array}$$

where the initial weight *W*_*D*_ is set as zero, diff is a function for calculating the absolute difference, and P(C) stands for the probability of this attribute appears in class C. This algorithm seeks *M* for each different class and averages their contribution for updating estimates *W*  _*D*_, which estimates the ability of features for the target values. In order to reduce useless features from the data set, this study applies the RELIEF-F algorithm to calculate the weight values of each attribute in the data set and then discards all features with negative weight values for reducing the dimension of feature vectors. Besides that, RELIEF-F originally needs to search P nearest neighbors from the same class. The number P requires to be defined by the user. In this study, P is defined as 10 based on the reference paper [33]. In our proposed model, RELIEF-F is used before starting training model. It is an efficient algorithm for reducing dimension of features and complex computation in the further process.

RELIEF-F algorithm using filter-method approach calculates a score (weight) for each feature to identify which features are most relevant to the set of instances. A weight is linked to each attribute, where the most relevant attribute has the highest weight. If a feature value difference is observed in a neighboring instance pair with the same class, the weight decreases. Alternatively, if a feature value difference is observed in a neighboring instance pair with different class values, the weight increases. Compared to positive weight features, negative weight features will have more chance in the same or closed class [41]. Moreover, Kira and Rendell demonstrated that, statistically, the relevance level of a relevant feature was expected to be larger than zero and that of an irrelevant one was expected to be zero or negative [32]. Therefore, generally, the threshold of RELIEF-F (*τ*) should be defined such as *τ* > 0.

### 3.3. Sample Selection Methods

To overcome the random selection limitation of Reduced-KELM and to enhance classification performance, this study proposes a novel sample selection method to replace the random selection part of model Reduced-KELM. This new method applies modified K-means and correlation detection selection methods to select the efficient samples. Before introducing the proposed approach, we described the two new sample selection methods.

#### 3.3.1. K-Means

K-means [42] is a classical clustering method, which solves the optimal clustering center and optimal classification by learning. It has a high learning efficiency and can process large-scale data [43]. In this paper, the K-means algorithm clusters the data for achieving stable prediction and higher accuracy than the conventional Reduced-KELM.

K-means is an unsupervised learning clustering algorithm and one of the most popular clustering algorithms at present. It applies the Euclidean distance metric as the standard similarity analysis method and divides the whole into a certain number of classes with high similarity. It assists in decreasing the number of samples and applying the cluster centroid position to stand for the original samples. The main goal of the K-means algorithm is to minimize the sum of the squared errors on all *Z* clusters. Its mathematical equation is as follows:

(14) $$\begin{array}{c} J\left(z\right)=\sum_{x\in Z}{\left\|x_{i}-\mu_{z}\right\|}^{2} \end{array}$$

where *μ*_*Z*_ represents the average value of all data that belonged to cluster *Z* (*Z* =  {1,2,…, *Z*}).

It is assumed that the data set contains N sample data, and the number of clusters is set as *Z*. Firstly, *Z* observations are selected from the whole data and set as the cluster center of the initial partition. Secondly, according to the similarity measurement method, it computes the distances between the undivided sample data and each cluster center point. After that, it divides the observations that are closer to the cluster center into the corresponding cluster. Then, it calculates the sum of square error between the center position and the corresponding observations for all classes. With moving the cluster center, observations belonged to each class are redivided until there is no change in the sum of squared errors of class. Finally, K-means will return the center position of each cluster. Algorithm 3 summarizes the process of the K-means sample selection method. The returned values can be used to replace the random part of Reduced-KELM for achieving stable forecasting performance.

#### 3.3.2. Correlation Detection Selection Method

There are many samples for the different classes in the classification. Generally, these samples are not all useful for the training model. As compared to the K-means clustering for sample selection, this study proposes an efficient technique named as Correlation Detection Selection method (CDS). It mainly finds the correlation among samples and discards the samples with high correlation values. Discarding the samples with similar information not only plays a positive role in training the model, but also replaces unstable random parts in Reduced-KELM. It increases the classification performance.

The main idea of CDS is to select memory without the high correlation values from all training data observations. Firstly, we initialize the threshold of CDS as *δ*, and the initial memory (mem) is defined as the first observation in the training data (mem  =  *X*_1_). Secondly, this method calculates the average value of correlation between the coming sample (**X**_**i**_) and filtered memory (mem). The coming sample will add into the filtered memory when the average correlation value is smaller than the threshold of CDS (*δ*). In contrast, it will exclude the coming data from the filtered memory. Algorithm 4 shows the pseudocode of CDS.

#### 3.3.3. Reformed Sample Selection Method

In this section, a new sample selection method named Reformed Sample Selection Method (RSSM), which applies the advantage of K-means and CDS to seek the more suitable samples for calculating kernel matrix, is proposed.

In RSSM, randomly set Cent as the initial centroids from the input matrix *X*_*L*_ and find out the samples that are nearest to each centroid based on Euclidean distance. Based on these samples, we can recalculate the position of centroids of Cent. Then, *J* can be computed. Cent can be computed until the value of *J* is not changed based on (14). In the next step, we initialize memory as mem  =  Cent_1_. Start from the second Cent, and calculate the average value of correlation (AC) between coming sample from Cent and mem. Based on the condition, mem can be updated. Finally, the matrix of mem can be returned. Algorithm 5 shows the detail about the pseudocode of Reformed Sample Selection Method.

### 3.4. Proposed Model: Reformed Reduced Kernel Extreme Learning Machine with RELIEF-F

For fair comparison and to further decrease the computation, the data is processed by RELIEF-F algorithm firstly. It is a first step to deal with input features. The output of RELIEF-F can be set as a new input data for the further steps. Based on the sample selection approaches, this study proposes a Reformed Reduced Kernel Extreme Learning Machine with RELIEF-F (R-RKELM), which employs the output of RSSM to replace the random selection part of Reduced-KELM. To prove the classification ability of the proposed model R-RKELM, it is compared with two other models, including Reduced Kernel Extreme Learning Machine with K-means and RELIEF-F (K-RKELM), and Reduced Kernel Extreme Learning Machine with Correlation Detection Selection and RELIEF-F (C-RKELM).

Firstly, based on RELIEF-F algorithm, the original features are processed. Then, the first model K-RKELM has applied the centroid positions of each cluster by K-means and all training samples to calculate the reduced kernel matrix. Model C-RKELM employs selected memory to replace the randomly selected data samples for developing conventional model Reduced-KELM.

The centroid positions of each cluster by K-means and the selected memory by CDS cooperated with the training samples to calculate the reduced kernel matrix, which replaces the kernel matrix calculated by random data samples in the conventional Reduced-KELM.

The proposed model applies the output of RSSM to replace the randomly selected samples in the conventional Reduced-KELM. RSSM is an unsupervised method. It mainly concentrates the training samples by K-means and obtains the centroid positions. And then, it applies CDS to discard the elements of the centroid positions with the high correlation value. The final remaining output replaces the random samples of conventional Reduced-KELM.

The pseudocode of R-RKELM is shown in Algorithm 6.

## 4. Experimental Works

To enhance the ability of classification and overcome the limitation of Reduced-KELM, this section designs two experiments. They employed the eight data sets, including benchmarks and real-world human activity data, to evaluate the classification ability for the RELIEF-F algorithm and Reduced-KELM with the different sample selection methods, respectively. This section mainly introduces data description, experimental design, and parameter setting. Lastly, based on the experimental design, the experimental results and discussion will be introduced.

### 4.1. Data Description

In the experimental section, the five benchmarks data sets and three human activity data sets are used for evaluating the classification ability.

A set of commonly used benchmarks includes German, Image, Ringnorm, Twonorm, and Waveform, available at UCI Machine Learning Repository [44]. These data sets contain binary class classification tasks.

Furthermore, with the data explosion and popularity of portable devices, researchers and developers pay more attention to human activity recognition, such as fall detection and sports detection in portable devices. Then, this study employs three real-world data sets to evaluate, including the Human Activities and Postural Transitions Recognition using Smartphone Data (HAPT) [45], Human Activity Recognition Using Smartphones Data Set (HARUS) [46], and Smartphone Data set for Human Activity Recognition in Ambient Assisted Living Data Set (Smartphone).

Besides, we separated the percentage of training and testing data in all benchmarks as 70% and 30%, respectively. The training and testing data of all real-world data sets are divided by their data source. We used the same division in our experiments. These data sets involve multiclass classification tasks.  Table 2 shows the details of each data set.

### 4.2. Experiment Design and Parameter Setting

To evaluate the ability of our proposed methods and compared models fairly, this study designs two experiments. All experiments are simulated on Matlab2014a in the laptop with Windows 10, 16 GB RAM environment.

The first experiment compares the classification performance of model Reduced-KELM with RELIEF-F algorithm with that of the conventional Reduced-KELM. It indicates the role the RELIEF-F algorithm plays in the features dimension reduction in Reduced-KELM. The performances of all benchmarks and human activity data in model Reduced-KELM are compared with those of Reduced-KELM with the RELIEF-F algorithm. The main aim of the RELIEF-F algorithm is to rank the features based on their importance in the classes and keep reliable attributes for the following training phase. Based on this algorithm, the feature selection process not only improves the classification performance, but also decreases the training time rather than conventional Reduced-KELM. To compare models fairly, the design of parameter setting needs to make sure that every model has the best performance under specific parameter setting. In the first experiment, the number of P nearest neighbors needs to be defined, which is critical to the performance of RELIEF-F algorithm. Based on the conclusion of paper [33], P is defined as ten in the first experiment. At the same time, we set the percentage of random selection as ten for all models in the first experiment, including the conventional Reduced-KELM and Reduced-KELM with RELIEF-F. Because the reference paper [8] concluded that Reduced-KELM randomly selected ten percentage of nodes that assisted on rapidly decreasing the training time, the performance of Reduced-KELM obtained was almost at the same level as that of KELM. Besides, due to the implementation of the kernel method, the kernel parameter impacts the performance in classification. For fair comparison among the models, the value of the kernel parameter is defined as one for all models in the first experiment.

On the other hand, the second experiment mainly observes the role the three different sample selection methods played in classification by model Reduced-KELM. These three methods successfully replace the random part of Reduced-KELM, respectively. This experiment shows the superior ability of selecting samples in the different sample selection methods and the ability of reducing the complexity computation of training model. It compares the performance of the proposed model R-RKELM with the conventional Reduced-KELM, K-RKELM, and C-RKELM. To reflect the connection between the first experiment and the second experiment, the second experiment applies the data sets that are processed by RELIEF-F algorithm. The parameters of RELIEF-F algorithm and kernel method in the second experiment are the same as the first one. To exhibit the performances of model under the different measurements, except for the common measurement accuracy and the corresponding Standard Deviation (SD) and Time, Sensitivity, Specificity, and Precision are employed to evaluate the performance in all experiments as well. At the same time, to observe the generalization ability, the fifty times will be run, and then calculate their average values of measurements when the model has a random selection method. A high standard deviation indicates that the accuracy values among fifty times are spread out over a wider range, and vice versa.

### 4.3. Experimental Results and Discussion

**Figure 2:**
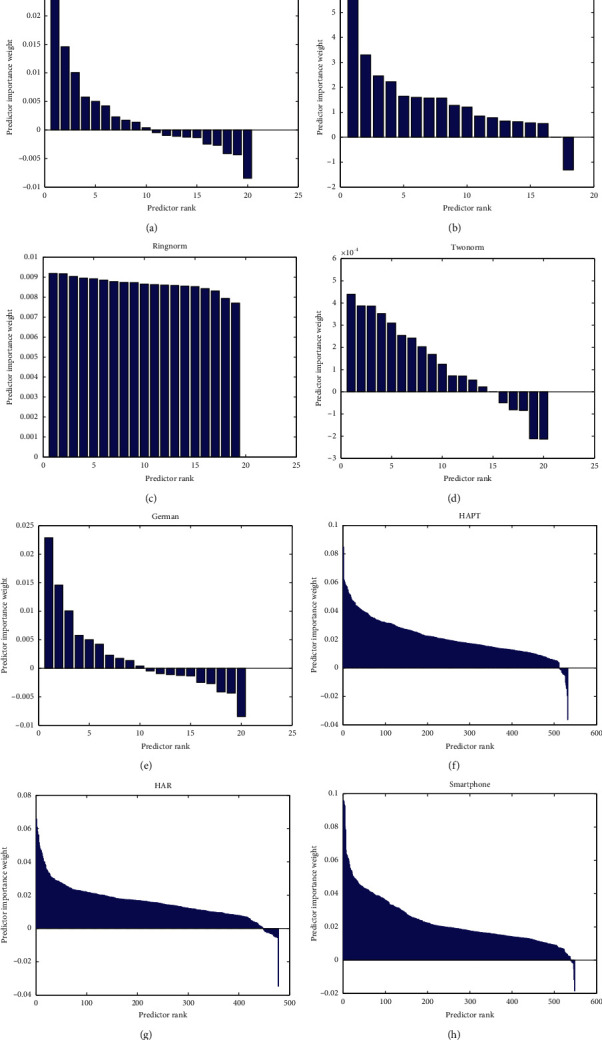
The distribution of predictor importance weights in features.

The first experiment demonstrates the differences between the conventional Reduced-KELM and Reduced-KELM with RELIEF-F algorithm (Relief-F). Relief-F algorithm is applied in the benchmarks and real-world data sets. The ranking of predictor weights is shown in Figure 2, which represents the level of importance of features.

Based on these bar charts in Figure 2, the values of the vertical axis represent the level of importance for features. Because of the conclusion from paper [32], compared to the positive weight features, the negative weight features have more chance in the same or closed class. These features probably are redundancy. And paper [41] showed that the features with positive weights have much better performance than that with negative weights. Therefore, this study discards the features with values that are below zero.

The dimension of data sets can be reduced by RELIEF-F algorithm. The final dimension of each data set is shown in Table 3. The column named ‘Difference' represents the number of features that the RELIEF-F algorithm has reduced. For example, the German data filters half of the features from original data, and Ringnorm has only reduced one attribute by RELIEF-F.

Table 4 indicates the performances between Reduced-KELM and Reduced-KELM with RELIEF-F in the eight data sets. It represents the performance of accuracy, Difference (Accuracy of Reduced-KELM - Accuracy of Relief-F), Standard Deviation (SD), and training time (Time). Besides, the other three measurements are also shown in Table 4, including Sensitivity, Specificity, and Precision.

In terms of accuracy, only one data set (Twonorm) shows the best performance in model Reduced-KELM. The rest of the data sets obtain the super classification ability in the Relief-F model rather than the conventional Reduced-KELM. On average, the growth rate of accuracy by relief-F in these data sets reaches 1.33 %. At the same time, the positive value of difference indicates that the super classification ability in model relief-F is better than the conventional model Reduced-KELM, and vice versa. The maximum difference of accuracy appears in German data. On the contrary, the image obtains the minimum difference. Although three data sets (including Twonorm, Waveform, and HAPT) have the same performance in SD for the conventional Reduced-KELM, Relief-F obtains the minimum value in standard deviation for the rest of data sets. In aspect of training efficiency, the main achievement of Relief-F is saving the training time. Especially for the data with high dimensions, such as HAPT, HARUS, and smartphones, the training time (minutes) is reduced by relief-F with 0.0371, 0.1102, and 0.031, respectively. In other measurements, the Relief-F algorithm has reduced Sensitivity for the majority of the data sets. This situation indicates that the Relief-F algorithm has a more stable prediction ability than the conventional Reduced-KELM. At the same time, the same performances appear in Specificity and Precision. Expect for Twonorm, HAPT, and HARUS, the Relief-F algorithm shows much better classification performance than the Reduced-KELM model. Therefore, the Relief-F method not only improves the accuracy of classification in benchmarks and real-world data sets, but also has saved the training time.

The second experiment compares the performance in classification of the proposed model R-RKELM with the model K-RKELM and C-RKELM.  Table 5 collects the information about accuracy, SD, time, sensitivity, specificity, and precision for the model K-RKELM, C-RKELM, and R-RKELM.

In terms of accuracy, the proposed model, R-RKELM, successfully assists on enhancing classification performance in model Reduced-KELM. For the whole data sets, model R-RKELM obtains the best performance in accuracy than the other two models. Moreover, due to the random characteristic in the K-means algorithm, there is a minor difference in performance appearing in forecasting results when K-RKELM and R-RKELM are run repeatedly. The standard deviation represents the degree of forecasting difference in all predictions. Except for HARUS and Smartphone data, model R-RKELM obtains the lowest value in Standard Deviation. In the aspect of training time, benchmarks data sets take longer training time in model C-RKELM than model R-RKELM. In HAPT and HARUS data, model R-RKELM takes the similar time in the training process than model C-RKELM. Model R-RKELM in Smartphone data takes less than five times as much training time as model C-RKELM. In Sensitivity, model C-RKELM has the best performance than other models. Model R-RKELM shows the best performance in Specificity for the majority of data sets. Compared with performance in Precision, only real-world data sets achieve the best performances in model R-RKELM. However, there is a small gap between R-RKELM and other models. Therefore, the three sample selection methods play a positive role in Reduced-KELM for enhancing classification performance. And the proposed model R-RKELM has the best achievement in terms of classification performance.

## 5. Statistical Analysis

According to the comparison results in Tables 4 and 5, the best performances are achieved by model Relief-F and R-RKELM, respectively. To measure the level of classifying ability between R-RKELM and Relief-F, this study has applied Wilcoxon-signed Rank Test to test whether R-RKELM has superior in classification ability to Relief-F.

Table 6 reports the accuracy of R-RKELM and Relief-F in each data set. The difference between these two models in terms of accuracy for all the eight data sets is computed. The ranking number based on these absolute difference values is shown. Then, values of *R*+ and *R*− are computed. *R*+ represents the sum of ranks for the positives, and *R*− stands for the sum of ranks for the negatives in Table 6. *R*+ is 36 and *R*− is 0. Based on the table of critical values, at the confidence level of *p* = 0.05, the difference between the algorithms is significant if the value of *R*− is less than 3. Based on the result, we conclude that model R-RKELM has the super classification ability with model Relief-F statistically.

## 6. Conclusion

This study introduces a novel classifier called Reformed Reduced Kernel Extreme Learning Machine with RELIEF-F (R-RKELM) for human action recognition. The proposed framework has two stages. In the first stage, it employs RELIEF-F to discard the irrelevant features with the negative values in the weight. The second stage focuses on the training samples selection for the reduction of computation complexity. Moreover, the proposed approach NSSM in R-RKELM takes advantage of K-means and CDS to replace the randomly reduced part of conventional Reduced-KELM, which reduces the unstable element for classification. Based on the experimental evaluation on eight data sets and statistical analysis, R-RKELM has much better performance in terms of classification and training time than conventional Reduced-KELM than other baselines. The accuracy of the proposed model reached around 90 %. In the future, we will focus on the parameter dependency in our proposed model. The kernel parameter impacts the performance of classification.

## Figures and Tables

**Algorithm 1:**
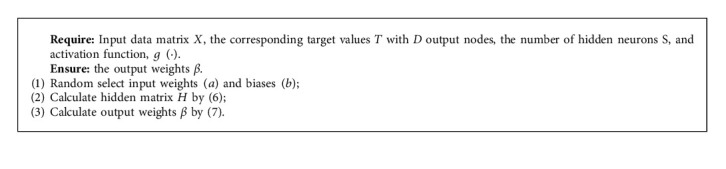
The training process of ELM.

**Algorithm 2:**
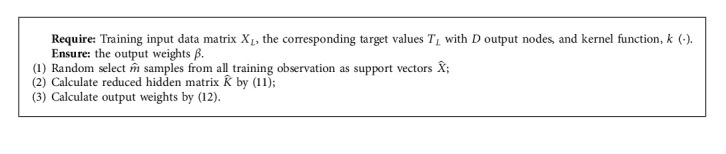
The training process of Reduced-KELM.

**Algorithm 3:**
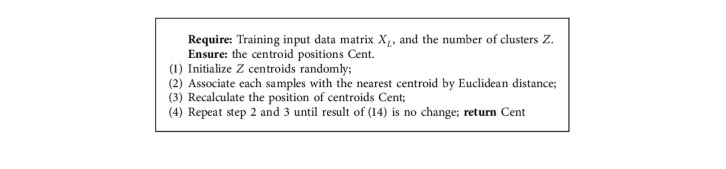
The process of K-means.

**Algorithm 4:**
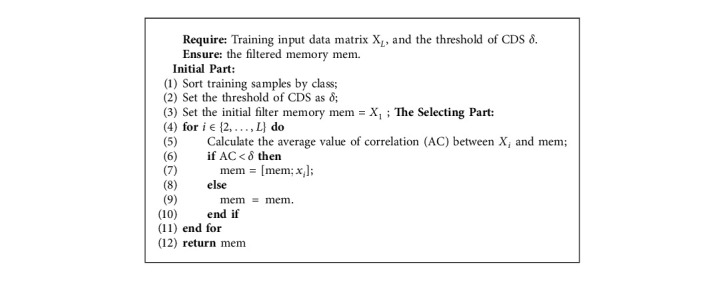
The process of Correlation Detection Selection method.

**Algorithm 5:**
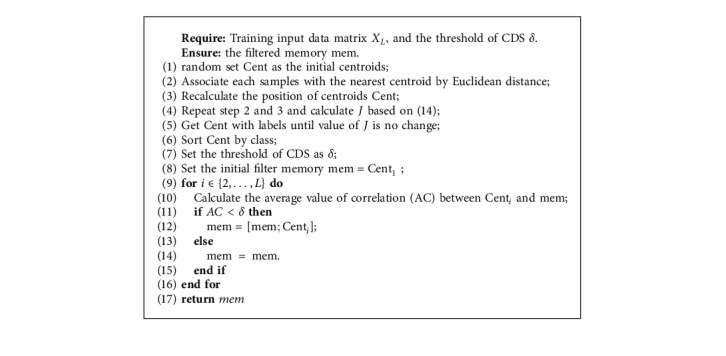
The process of Reformed Sample Selection Method.

**Algorithm 6:**
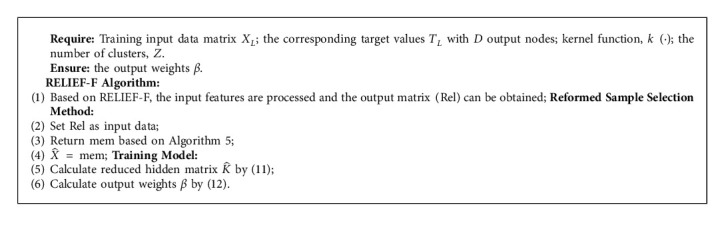
The training process of R-RKELM.

**Table 1:** The advantages and drawbacks of ELM and variant ELM models.

Model	Advantages	Drawbacks
ELM	Fast training speed; same or better performance than the conventional models	Unstable forecasting performance; parameter dependency (2 parameters)
KELM	Stable performance	Heavy computation; parameter dependency (1 parameter)
Reduced-KELM	Less computation than KELM	Unstable forecasting performance; parameter dependency (1 parameter)

**Table 2:** Details of Data sets.

Data	Training samples	Testing samples	Feature	Class
German	700	300	20	2
Image	1460	626	18	2
Ringnorm	5180	2220	20	2
Twonorm	5180	2220	20	2
Waveform	3500	1500	21	2
HAPT	7767	3162	561	6
HARUS	7352	2947	561	6
Smartphone	4252	1492	561	6

**Table 3:** The reduction of features dimension by RELIEF-F.

Data	Feature	Dimension after RELIEF-F	Difference
German	20	10	10
Image	18	16	2
Ringnorm	20	19	1
Twonorm	20	14	6
Waveform	21	10	11
HAPT	561	532	29
HARUS	561	478	83
Smartphone	561	548	13

**Table 4:** The performances of Reduced-KELM and Relief-F.

Data	Model	Accuracy (%)	Difference	SD	Time	Sensitivity	Specificity	Precision
German	Reduced-KELM	71.53	8.74%	0.06	0.0013	0.5790	0.4962	0.5175
	Relief-F	**80.27**		**0.03**	**0.0012**	0.5164	0.5108	0.5068

Image	Reduced-KELM	85.70	0.16%	0.03	0.0086	0.8623	0.8510	0.8555
	Relief-F	**85.86**		**0.02**	**0.0063**	0.8340	0.8780	0.8815

Ringnorm	Reduced-KELM	60.06	0.85%	0.02	0.1128	0.5961	0.6079	0.7786
	Relief-F	**60.91**		**0.01**	**0.1031**	0.6135	0.6075	0.7786

Twonorm	Reduced-KELM	**94.10**	-1.65%	0.01	0.1022	0.9401	0.9377	0.9115
	Relief-F	92.45		**0.01**	**0.0934**	0.9245	0.9216	0.8853

Waveform	Reduced-KELM	84.29	0.93%	0.01	0.0474	0.8324	0.8090	0.8148
	Relief-F	**85.22**		**0.01**	**0.0384**	0.8169	0.8223	0.8160

HAPT	Reduced-KELM	88.52	0.95%	0.08	0.4155	0.9580	0.9434	0.8731
	Relief-F	**89.47**		**0.07**	**0.3753**	0.9552	0.8936	0.7479

HARUS	Reduced-KELM	84.02	5.66%	0.07	0.3826	0.9470	0.9419	0.8643
	Relief-F	**89.68**		**0.06**	**0.3589**	0.9410	0.8851	0.7623

Smartphone	Reduced-KELM	85.52	1.03%	0.07	0.1261	0.7749	0.7535	0.7280
	Relief-F	**86.55**		**0.07**	**0.0951**	0.7869	0.7926	0.7532

**Table 5:** The performance between relief-F and RKELM with two sample selection methods.

Data	Model	Accuracy (%)	SD	Time	Sensitivity	Specificity	Precision
German	K-RKELM	83.53	0.01	0.0011	0.5531	0.5881	0.6045
	C-RKELM	81.67	0.02	0.0419	0.9157	0.1538	0.8787
	R-RKELM	**85.44**	0.01	0.0056	0.6753	0.3940	0.7184

Image	K-RKELM	87.43	0.01	0.0063	0.8694	0.8736	0.8816
	C-RKELM	87.70	0.00	0.2610	0.9634	0.7819	0.8294
	R-RKELM	**87.75**	0.00	0.2536	0.9040	0.8424	0.8700

Ringnorm	K-RKELM	55.32	0.00	0.1265	0.5556	0.5541	0.7634
	C-RKELM	67.66	0.00	10.2167	1.0000	0.3555	0.6064
	R-RKELM	**69.37**	0.00	6.1088	0.7986	0.5910	0.7454

Twonorm	K-RKELM	94.52	0.00	0.1482	0.9382	0.9393	0.9195
	C-RKELM	95.23	0.00	9.1533	0.9385	0.9566	0.8735
	R-RKELM	**95.35**	0.00	5.7655	0.9479	0.9539	0.9096

Waveform	K-RKELM	85.51	0.01	0.0305	0.8454	0.8258	0.8298
	C-RKELM	85.47	0.00	3.7682	0.8757	0.8117	0.9045
	R-RKELM	**85.84**	0.00	1.6029	0.8594	0.8344	0.8597

HAPT	K-RKELM	89.50	0.09	0.3997	0.9709	0.8914	0.7367
	C-RKELM	90.58	0.06	18.8531	0.8932	0.8309	0.7481
	R-RKELM	**92.87**	0.06	24.1748	0.9760	0.9197	0.7869

HARUS	K-RKELM	89.79	0.08	0.3253	0.9618	0.8945	0.7552
	C-RKELM	88.83	0.00	20.1623	0.8468	0.8898	0.6087
	R-RKELM	**92.81**	0.05	20.1696	0.9649	0.9221	0.8029

Smartphone	K-RKELM	86.68	0.06	0.2197	0.8055	0.7888	0.7092
	C-RKELM	86.68	0.00	5.6465	0.7945	0.8057	0.7178
	R-RKELM	**86.92**	0.06	1.3800	0.8126	0.8090	0.7268

**Table 6:** The Wilcoxon signed-rank test between R-RKELM and Relief-F.

Data	Model		Difference	Signed	Rank
	R-RKELM (%)	Relief-F (%)			
German	85.44	80.27	5.17	+	7
Image	87.75	85.86	1.89	+	3
Ringnorm	69.37	60.91	8.46	+	8
Twonorm	95.35	92.45	2.90	+	4
Waveform	85.84	85.22	0.64	+	2
HAPT	92.87	89.47	3.40	+	6
HARUS	92.81	89.68	3.13	+	5
Smartphone	86.92	86.55	0.37	+	1

## Data Availability

All data sets used in paper are from UCI Machine Learning Repository
